# Peroxidasin contributes to lung host defense by direct binding and killing of gram-negative bacteria

**DOI:** 10.1371/journal.ppat.1007026

**Published:** 2018-05-18

**Authors:** Ruizheng Shi, Zehong Cao, Hong Li, Jochen Graw, Guogang Zhang, Victor J. Thannickal, Guangjie Cheng

**Affiliations:** 1 Division of Pulmonary, Allergy and Critical Care Medicine, Department of Medicine, University of Alabama at Birmingham, Birmingham, AL, United States of America; 2 Helmholtz Center Munich, German Research Center for Environmental Health, Institute of Developmental Genetics, Neuherberg, Germany; 3 Department of Cardiovascular Medicine, Xiangya Hospital, Central South University, Changsha, China; University of Michigan Medical School, UNITED STATES

## Abstract

Innate immune recognition is classically mediated by the interaction of host pattern-recognition receptors and pathogen-associated molecular patterns; this triggers a series of downstream signaling events that facilitate killing and elimination of invading pathogens. In this report, we provide the first evidence that peroxidasin (PXDN; also known as vascular peroxidase-1) directly binds to gram-negative bacteria and mediates bactericidal activity, thus, contributing to lung host defense. PXDN contains five leucine-rich repeats and four immunoglobulin domains, which allows for its interaction with lipopolysaccharide, a membrane component of gram-negative bacteria. Bactericidal activity of PXDN is mediated *via* its capacity to generate hypohalous acids. Deficiency of PXDN results in a failure to eradicate *Pseudomonas aeruginosa* and increased mortality in a murine model of *Pseudomonas* lung infection. These observations indicate that PXDN mediates previously unrecognized host defense functions against gram-negative bacterial pathogens.

## Author summary

Multicellular organisms have evolved diversified host defense mechanisms for survival against invading pathogens. Of the mechanisms, the recognition of pathogens is classically mediated by the interaction of host and pathogen, which triggers a series of downstream responses to eliminate pathogens. Proteins that both selectively and directly interact with and kill pathogens are not well identified. In current study, we have determined the dual-function mechanisms of PXDN -mediated bacteria killing. We provide the first evidence for a novel role of PXDN in directly binding to gram-negative bacteria and mediating bactericidal activity. PXDN is highly expressed in the lung and secreted into epithelial lining fluid of the lung, and is induced by LPS and TNF-α. PXDN mutant mice reveal impaired lung host defense in acute lung infection model of *P*. *aeruginosa*. PXDN is a new class of bactericidal enzyme with dual function of recognizing and killing pathogens. This finding of an enzyme with dual function has important implications for new conceptual understanding of the innate immunity as well as for therapeutic development.

## Introduction

The lung is exposed to a constant barrage of inhaled harmful agents and microorganisms. Several layers of defense in the normal lung help prevent infection from inhaled or aspirated microorganisms. These include the mechanical filtering of particles that occur in the nasal airway, the trapping of particles in mucus and mucociliary clearance. Respiratory epithelial cells also secrete surfactant proteins, antimicrobial peptides and complements; all of these secreted proteins are important in innate immunity [1, 2]. In addition, alveolar macrophages, neutrophils, lymphocytes and circulating antibodies participate in the clearance of microorganisms from the lung [3].

Innate immune responses are classically initiated by recognition of pathogens through host pattern-recognition receptors (PRRs) [4, 5]. The interaction between PRRs and the specific pathogen-associated ligands, named pathogen-associated molecular patterns (PAMPs) activates the downstream signaling events and host defense mechanisms to eliminate invading pathogens [4, 5]. Recognition of the PAMPs allows the host immune system to distinguish infectious pathogens from the host. Four families of PRRs have been identified, the Toll-like receptors (TLRs), nucleotide-binding and oligomerization domain (NOD)-like receptors (NLRs), retinoic acid inducible gene-1 (RIG-1) like receptors (RLRs) and C-type lectin receptors (CLRs) [6]. Members of these families contain at least one of nine highly conserved protein domains such as leucine-rich repeats (LRRs) and immunoglobulin-like (Ig) domains [6]. These domains are crucial for recognizing PAMPs within invading pathogens. Common bacterial PAMPs include lipopolysaccharide (LPS), peptidoglycan (PGN), bacterial flagellin and lipoteichoic acid [7].

The heme-containing peroxidase (hPx) family is known to participate in host defense [8, 9]. Myeloperoxidase (MPO), the proto-enzyme of hPx family, has been extensively investigated [8, 9]. MPO was thought to be the only peroxidase capable of generating HOCl under physiological conditions [8]. It has been proposed that MPO binds to pathogens as a result of its higher cationic surface charge (pI = 9.3) in the acidic environment of the phagosome [10, 11]; however, this interaction is non-specific and any molecule with anionic surface charge, including but not limited to pathogens, may potentially interact with MPO with its attendant risk of collateral damage. Lactoperoxidase (LPO) is secreted into some body fluids including milk, saliva and mucus of airway, but not plasma and alveolar lining fluid; its physiological role is to prevent microbial growth at these mucosal surfaces [12, 13]. However, the presence of hPx with bactericidal activity in the lower airways and alveolar regions has not been demonstrated.

**Fig 1:**
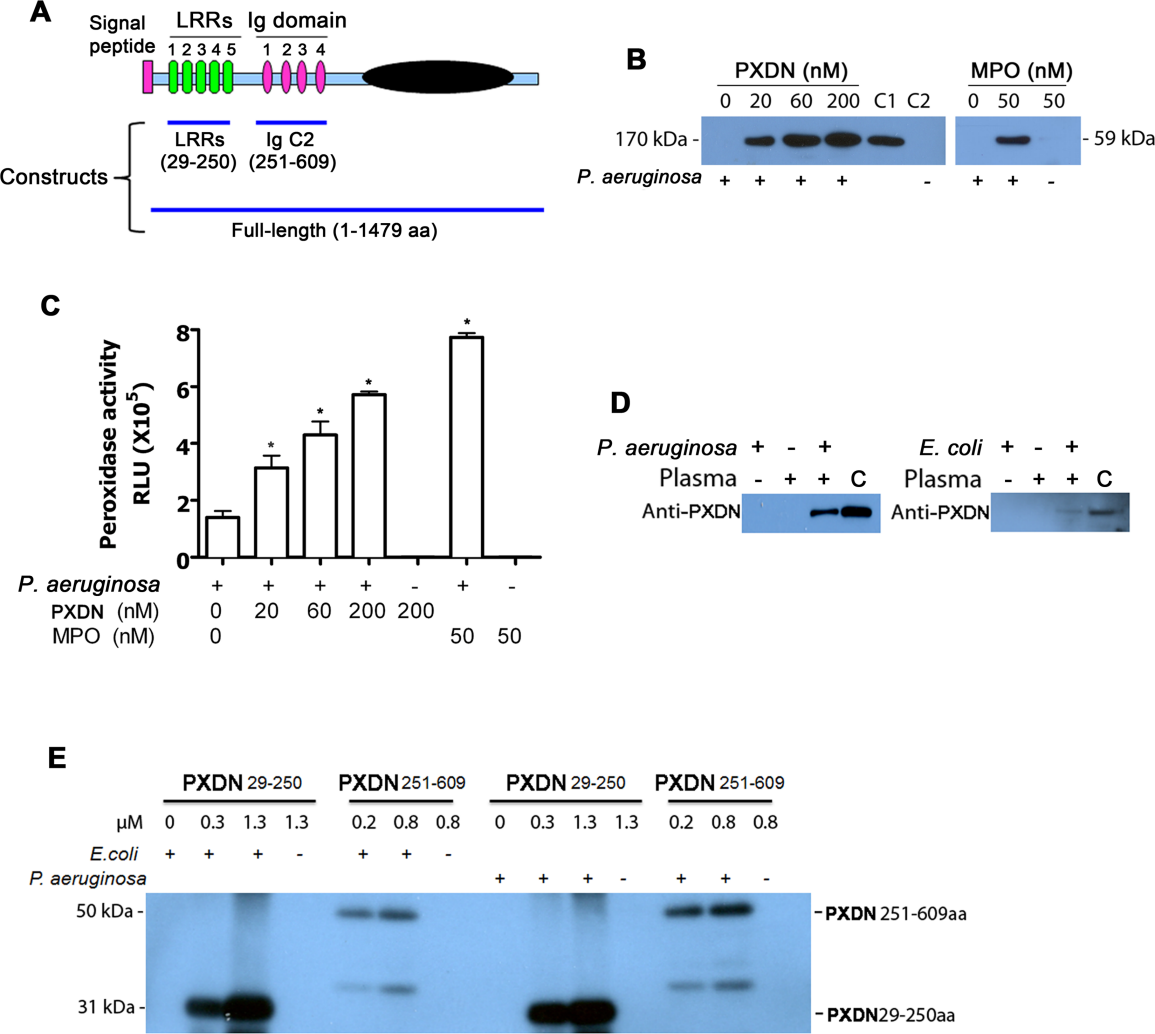
PXDN binds to GN bacteria. **(A)** Diagram of human PXDN and recombinant proteins of PXDN used in the study. All constructs for preparation of recombinant proteins of PXDN were polyhistidine-tagged either cloned in plasmid pET30 or pcDNA3.1. The subcloned region and related domain structure are indicated. **(B)** *P*. *aeruginosa* binds to full-length PXDN. *P*. *aeruginosa* strain K was added to PBS containing PXDN or MPO as indicated. The mixtures were incubated at 37°C for 1 h. *P*. *aeruginosa* mixtures were centrifuged at 3099 x g for 5 min. The pellets were washed twice. One-half of the bacterial suspension was subjected to immunoblotting using anti-His antibody (left panel) or anti-MPO (right panel) antibody. C1 was PXDN positive control which was directly loaded. C2 was negative control in which 200 nM PXDN did not mixed with bacteria; it was centrifuged at 3099 x g for 5 min as experimental groups. **(C)** The remaining bacterial suspension in “B” was subjected to peroxidase activity assay using L-012 as chemiluminescent substrate. Note: MPO is known to bind to *P*. *aeruginosa via* its cationic charge, and served as a positive control in these experiments. *P<0.001 *vs*. bacteria only. Data are the representatives of at least three independent experiments. **(D)** *E*. *coli* and *P*. *aeruginosa* bind to plasma PXDN. 4 x 10^8^ of *E*.*coli* K12 or *P*. *aeruginosa* strain K in 50 μL PBS were added to 50 μL human plasma. The mixtures were incubated at 37°C for 1 h. Bacterial suspensions were spun down at 3099 x g for 5 min, and then washed twice with 500 μL PBS. Samples were subjected to immunoblotting using anti-PXDN antibody. “C” represents positive control of 1 μL of plasma containing 200 ng of PXDN, which was directly loaded onto the gel. Data are the representatives of three independent experiments. **(E)** Truncated PXDN binds to live *P*. *aeruginosa* and *E*. *coli*. Recombinant truncated peptides of PXDN 29-250aa or PXDN 251-609aa were added to live bacterial suspensions of *P*. *aeruginosa* strain K and *E*. *coli* K12, respectively. The mixtures were incubated at 37°C for 1 h. *P*. *aeruginosa* and *E*. *coli* suspension were centrifuged at 3099 x g for 5 min, and washed twice with PBS. The bacterial lysates were subjected to immunoblotting using anti-His antibody and visualized by chemiluminescence. A mixture containing only 1.3 μM of recombinant PXDN peptide without bacteria served as negative control (to verify no binding of PXDN peptide to the test tube). Data are representatives of three independent experiments.

Vascular peroxidase-1 (VPO1) is a newly-identified member of the hPx family in mammals [14]. The ortholog of VPO1 in *Drosophila* is known as peroxidasin (PXDN; as accepted by HUGO Gene Nomenclature Committee) [15]. In this article, the official name of PXDN is used instead of the alias, VPO1. PXDN catalyzes generation of hypohalous acids and kills bacteria *in vitro* [16, 17]. Homozygous mutation in *PXDN* causes developmental defects including congenital cataract, corneal opacity and glaucoma [18], abnormalities which are also present in PXDN mutant mice [19]. Unlike the classic hPx’es (MPO, eosinophil peroxidase, LPO and thyroid peroxidase), the expressions of which are restricted to specific cells or tissues, PXDN is more ubiquitously expressed [14]. PXDN is found at high circulating levels in human and mouse plasma, approximately 1.1 μM and 2.6 μM, respectively [20]. We previously reported that PXDN is the second mammalian hPx capable of catalyzing the oxidation of chloride in the presence of H_2_O_2_ to generate HOCl [16]. Interestingly, PXDN is unique among members of the hPx family [14]; it has additional domains in its N-terminus that include five LRRs and four Ig C2 domains (Fig 1A). LRRs and Ig domains are highly conserved protein domains and important in innate immune pattern recognition. However, the physiological function of PXDN in host defense is unclear. In the current study, we have identified a novel dual-function activity of PXDN, with its N-terminus recognizing LPS and its C-terminus mediating bactericidal killing, utilizing both *in vitro* and *in vivo* approaches. This is the first study, to our knowledge, demonstrating a critical role for PXDN in host defense of the lung.

## Results

### PXDN binds to gram-negative (GN) bacteria

Our data reveal that PXDN is able to generate hypohalous acids and kills bacterial *in vitro* [16, 17]. However, the precise mechanism of bactericidal activity of PXDN is unknown. PXDN contains specific N-terminus with five LRRs and four Ig C2 domains, and is predicted in protein-protein interaction and/or protein-pathogen interaction. Its C-terminus, which mainly consists of the peroxidase domain, is responsible for the generation of hypohalous acids [14]. We hypothesize that the N-terminus of PXDN binds to GN bacteria and facilitates bacterial killing by the peroxidase activity at the C-terminus. We sub-cloned full-length PXDN (FL-PXDN) as well as specific constructs containing LRRs and/or Ig C2 domains of PXDN into expression plasmids harboring His-tag (Fig 1A). These recombinant proteins were expressed either in *E*. *coli* or human HEK293 cells, and purified using HisPur resin. We first mixed recombinant FL-PXDN with *Pseudomonas aeruginosa (P*. *aeruginosa)*; bacterial suspensions were spun down by centrifugation. PXDN was detected in the bacterial pellets (Fig 1B), and a dose-dependent increase of peroxidase activity was verified in these fractions (Fig 1C). Additionally, we assessed whether endogenous PXDN in circulating plasma from healthy human volunteers can bind to *P*. *aeruginosa* and *E*. *coli*. Live *P*. *aeruginosa* and *E*. *coli* bacterial suspensions were mixed with human plasma. After centrifugation, PXDN was co-precipitated with bacteria, suggesting direct interaction between bacteria and plasma PXDN (Fig 1D). Unlike MPO, the pI value of PXDN is near neutral (pI = ~7.0); thus, the interaction of PXDN with bacteria is unlikely to be due to its surface charge. Ceruloplasmin is a ferroxidase enzyme present in circulating plasma at a concentration of 20 to 60 mg/dL (1.5–4.5 μM) [21]. We utilized this enzyme as an internal control to verify the specificity of the interaction of bacteria with plasma PXDN. As expected, ceruloplasmin did not bind to *P*. *aeruginosa* while minimal binding to *E*. *coli* was detected (S1 Fig).

We further evaluated whether specific regions in PXDN mediates binding to *P*. *aeruginosa* and *E*. *coli*. Similarly, recombinant truncated peptides of PXDN 29-250aa and PXDN 251-609aa were incubated with bacteria, respectively. These recombinant peptides were able to bind to *P*. *aeruginosa* and *E*. *coli* in a dose-dependent manner (Fig 1E). The lower molecular weight bands (approximate 35 kDa) observed in the PXDN 251-609aa group likely represent degradation products of the PXDN 251-609aa peptide (Fig 1E). Together, these data demonstrate that PXDN interacts with GN bacteria *via* its N-terminus.

### PXDN binds to LPS

**Fig 2:**
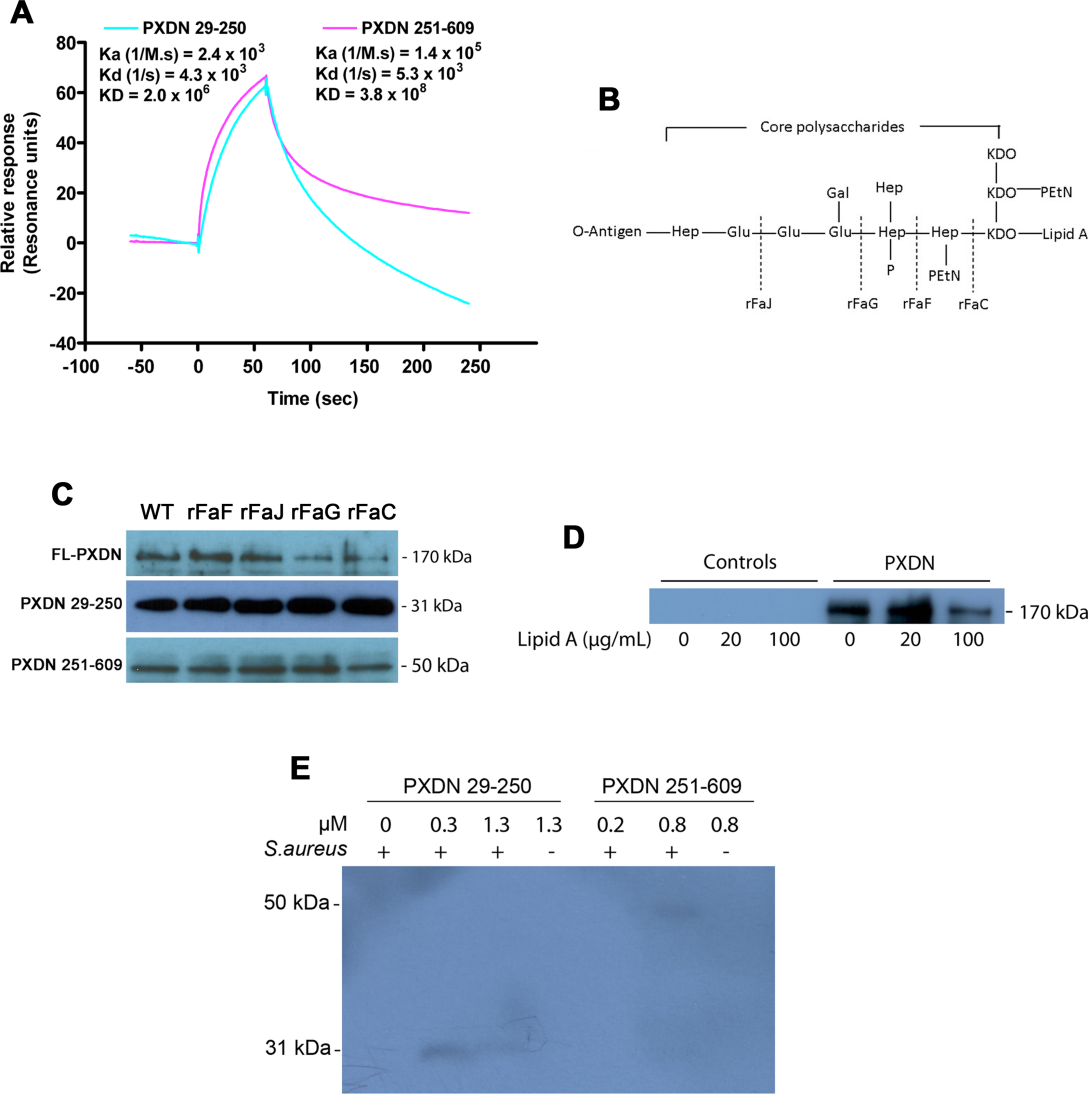
PXDN interacts with LPS. **(A)** PXDN binding to LPS determined by SPR. Recombinant peptides of PXDN 29-250aa or PXDN 251-609aa were immobilized on a NTA chip, and LPS (2 μM) was flowed over the chip. The binding data were collected and analyzed with Biacore T200 software. Data are the representatives of three independent experiments. **(B)** The typical structure of *E*. *coli* LPS and genes determining the biosynthesis of LPS. Dash indicates the stage point of LPS synthesis by the corresponding gene. KDO, 3-deoxy-D-manno-oct-2-ulosonic acid; Hep, L-glycero-D-manno-heptose; EtN, ethanolamine; Gal, D-galactose; Glu, D-glucose; P, phosphate. **(C)** Interaction of PXDN with LPS-deficient strains of *E*. *coli*. The binding experiments of truncated PXDN to LPS-deficient *E*. *coli* strains were carried out as in Fig 1B and 1E. 100 nM of FL-PXDN or truncated PXDN were utilized. *rFaF*, *rFaJ*, *rFaG* and *rFaC* are the LPS-deficient *E*. *coli* strains with mutation of related gene. “WT” is wild-type of *E*. *coli* K12 BW25113, which is the parent strain of LPS-deficient *E*. *coli* strains. Data are representatives of three independent experiments. **(D)** Lipid A inhibits PXDN binding to GN bacteria. Lipid A was mixed with *E*. *coli* K12 and PXDN similar to Fig 1B. The pellets were washed twice and subjected to immunoblotting using anti-His antibody. The data are representatives of two independent experiments. **(E)** The binding capacity of PXDN to GP bacteria is limit. Recombinant peptides of PXDN 29-250aa or PXDN 251-609aa were added to live bacterial suspensions of *S*. *aureus*, similar to Fig 1E. The bacterial lysates were subjected to immunoblotting using anti-His antibody and visualized by chemiluminescence. Data are representatives of three independent experiments.

Next, we determined the mechanisms of PXDN binding to *P*. *aeruginosa* and *E*. *coli*. LPS, cell-surface polysaccharide, maintains outer membrane integrity and mediates host-pathogen interactions. LPS comprises O-antigen, core polysaccharides and lipid A. LPS from *E*. *coli* and *P*. *aeruginosa* has the same general structure. Lipid A and the proximal region of oligosaccharides are relatively conserved, while O-antigen is highly variable in composition and structure [22]. We hypothesized that LPS serves as a PAMP that directly binds the LRRs and/or Ig domains of PXDN. To determine whether LPS binds to PXDN, we performed surface plasmon resonance (SPR) assays. As shown in Fig 2A, both the LRRs and Ig domains of PXDN were able to bind LPS. The association constants of LRRs and Ig domains of PXDN to LPS are 2.4 x 10^3^ and 1.4 x 10^5^, respectively.

We further queried which component of LPS is responsible for the interaction with PXDN. *E*. *coli* LPS structure is determined by a set of genes that encode biosynthesis of LPS components at various stages (Fig 2B) [23]. For example, a mutation in the *rFaC* gene causes formation of LPS consisting of only lipid A and 3-deoxy-D-manno-oct-2-ulosonic acid (KDO), with an absence of the O-antigen and most of the core polysaccharides. We carried out bacterial binding experiments of FL-PXDN and truncated PXDN with the various mutants of *E*. *coli* LPS. FL-PXDN and the truncations of PXDN 29-250aa and PXDN 251-609aa bound to several LPS variants (Fig 2C). Importantly, both full-length and truncated forms of VPO-1 were able to bind to the LPS variant that only contains lipid A (*rFaC* mutant). Lipid A was able to inhibit PXDN binding to *E*. *coli* (Fig 2D). Since gram-positive (GP) bacteria lack of LPS, we determined whether the N-terminus of PXDN binds specifically to GN bacteria, but not to GP bacteria. Binding assays with the GP bacteria, *Staphylococcus aureus* (*S*. *aureus*), were carried using truncated PXDN 29-250aa and 251-609aa (the experiment was performed concurrently with the studies of GN bacteria, as shown in Fig 1E). In contrast to *P*. *aeruginosa* and *E*. *coli*, the N-terminal domains of both LRRs and Ig domains did not bind to *S*. *aureus* (Fig 2E). Taken together, these data strongly support that the N-terminal domains of PXDN bind to LPS of GN bacteria.

### LPS activates PXDN

**Fig 3:**
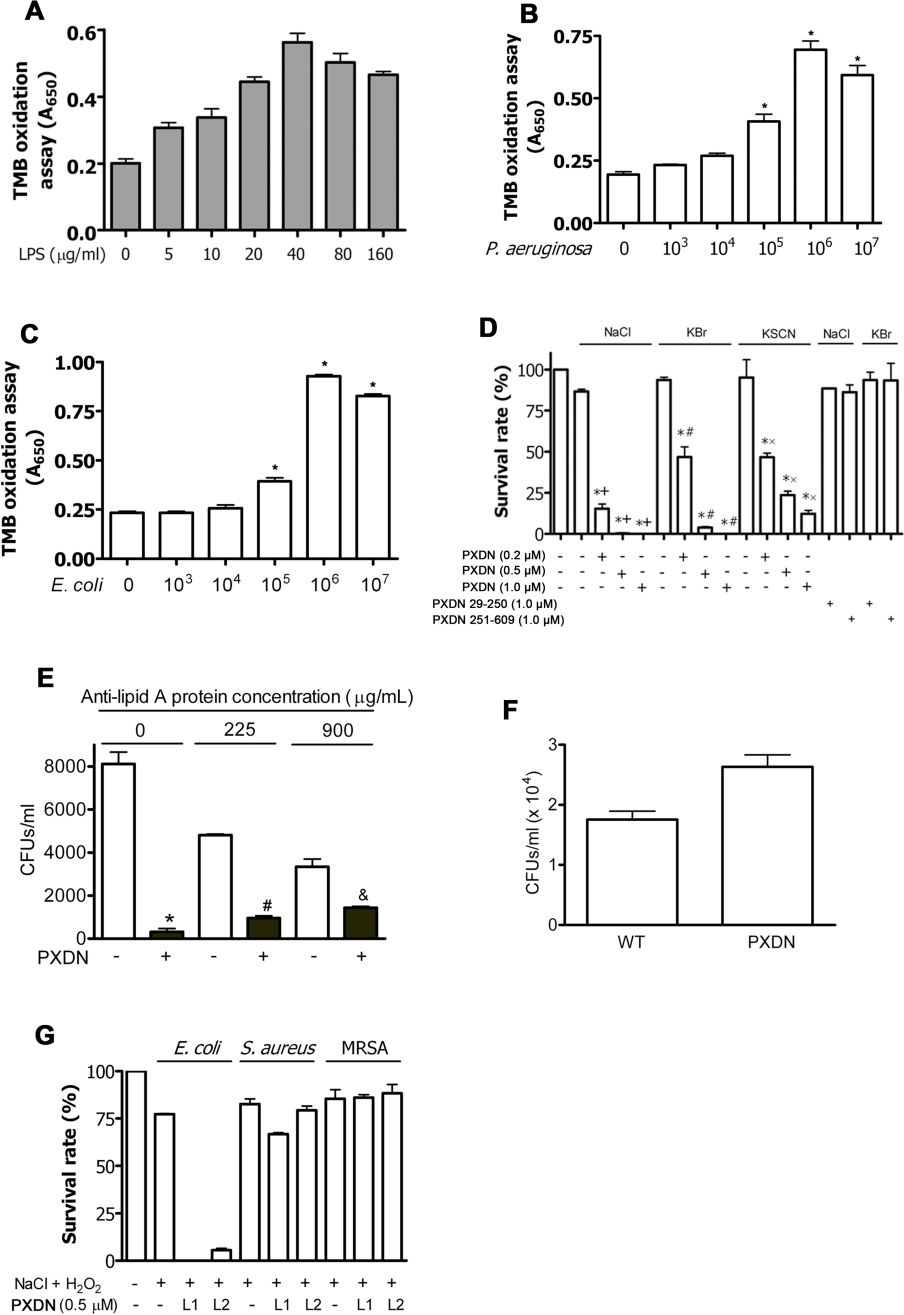
PXDN selectively kills GN bacteria *in vitro*. **(A)** LPS stimulates PXDN activity. Reaction mixtures (100 μL each) containing TMB solution, H_2_O_2_, recombinant FL-PXDN (400 nM/heme) and LPS as indicated were carried out at room temperature for 30 min. LPS and recombinant FL-PXDN were pre-incubated at 4°C for 30 min prior to mix with TMB solution. TMB oxidation was recorded at absorbance 650 nm. One-way analysis of variance, *P* < 0.001 for all comparisons. **(B)** Live *P*. *aeruginosa* stimulates PXDN activity. *P*. *aeruginosa* was pre-incubated with recombinant FL-PXDN at RT for 30 min in 20 mM of phosphate buffer without NaCl. The mixture was added into TMB solution (100 μL) and incubated at RT for 30 min. Absorbance at 650 nm was measured. *P<0.05 *vs*. non-bacteria control. **(C)** *E*. *coli* stimulates PXDN activity. The same experiment was carried out as in “B” where *P*. *aeruginosa* was replaced by *E*. *coli*. *P<0.05 *vs*. non-bacteria control. **(D)** PXDN kills GN bacteria *in vitro*. *P*. *aeruginosa* strain K suspensions were incubated in 50 mM phosphate buffer (pH 6.2) containing indicated amounts of recombinant FL-PXDN or 1 μM of truncated PXDN (PXDN 29-250aa and PXDN 251-609aa), 10 μM H_2_O_2_, and halide (140 mM NaCl, 100 μM KBr or 100 μM KSCN) at 37°C for 1 h. Cell mixtures were plated on LB agar plates and incubated at 37°C overnight. The control group contained *P*. *aeruginosa* only (lane 1). The CFUs were counted, and relative survival rates were calculated as CFUs in the experimental group divided by those in the control group. *P < 0.001 *vs*. Control; ^+^*P* < 0.001 *vs*. H_2_O_2_ + NaCl; ^#^*P* < 0.001 *vs*. H_2_O_2_ + KBr; ^x^*P* < 0.001 *vs*. H_2_O_2_ + KSCN. Data are representatives of three independent experiments. **(E)** Anti-lipid A antibody inhibits bacterial killing by PXDN. *E*. *coli* K12 was incubated in 50 mM phosphate buffer (pH 6.2) containing 2 μM recombinant PXDN, 10 μM H_2_O_2_, 140 mM NaCl and indicated amounts of anti-lipid A antibody, at 37°C for 1 h. Cell mixtures were plated on LB agar plates and incubated at 37°C overnight. The colonies were counted. Paired Student’s *t*-test: *P = 0.0031; #P = 0.0016; &P = 0.0464. **(F)** Sera from PXDN-deficient mice are impaired to kill bacteria. Sera from C57BL/6 and PXDN-deficient mice were used in the experiments. 100 μL reaction mixtures contained 50 μL serum and 50 μL PBS containing *P*. *aeruginosa* strain K and 50 μM H_2_O_2_. After incubation at 37°C for 1h, the mixture was plated on LB agar plates and incubated at 37°C for overnight. n = 3. P = 0.03. **(G)** PXDN cannot kill GP bacteria. The bacterial killing experiment was carried out similar to “D” in the presence of H_2_O_2_ and NaCl. Two lots of recombinant PXDN were utilized (L1 and L2). *E*. *coli* K12 was used as positive control. One-way analysis of variance, *P* < 0.001 for *E*. *coli*; P = 0.1308 for *S*. *aureus*; P = 0.6460 for MRSA. All data are representatives of at least three independent experiments.

We explored whether bacterial binding induces PXDN activation. LPS was added to reaction mixtures containing PXDN, H_2_O_2_ and 3,3’,5,5’-tetramethylbenzidine (TMB), and oxidation products were measured as an index of PXDN-dependent peroxidase activity. PXDN activity increased in a dose-dependent manner with the addition of LPS (Fig 3A). Maximal PXDN activity occurred at an LPS concentration of 40 μg/mL; the induced activation was ~2.8 fold higher than control without LPS (Fig 3A). Higher concentrations of LPS inhibited PXDN activity (Fig 3A). Further, live *P*. *aeruginosa* and *E*. *coli* at varying colony formation units (CFUs) were added to the reactions. Stimulation of PXDN activation was found to be dependent on the number of CFUs, with PXDN activity increasing approximately 3.7 and 4.2 folds by *P*. *aeruginosa* and *E*. *coli*, respectively, at 10^6^ CFUs (Fig 3B and 3C). Live bacteria in the absence of PXDN are incapable of oxidizing TMB (S2 Fig). Interestingly, live *E*. *coli* did not activate MPO and LPO, which are not known to have specific interacting domains with LPS or bacteria (S3A and S3B Fig). Thus, both LPS and live bacteria are capable of activating PXDN, but not MPO or LPO.

### PXDN kills GN, but not GP bacteria

Since the hPx family plays an important role in host defense [8, 9], we further investigated whether PXDN in the presence of H_2_O_2_ is able to kill bacteria, similar to the MPO/H_2_O_2_ system in phagocytes [8, 24] and the LPO/H_2_O_2_ system at mucosal surfaces [25]. Bacterial suspensions of *P*. *aeruginosa* were incubated with H_2_O_2_ and halide anion (chloride, bromide or thiocyanate, a pseudohalide), in the absence or presence of PXDN (0.2 to 1 μM). Bactericidal activities were assessed by survival rates of *P*. *aeruginosa* relative to control (without PXDN). Survival of *P*. *aeruginosa* was dose-dependent, with 0.2 μM PXDN inducing ≥ 50% killing effect under these conditions (Fig 3D). Physiological concentrations of PXDN (~1.1 μM) [20] completely killed the bacteria in the presence of physiological concentrations of Cl^-^ or Br^-^ and 10 μM H_2_O_2_ (Fig 3D). PXDN was less efficient in killing *P*. *aeruginosa* in the presence of 10 μM H_2_O_2_ and 100 μM of SCN^-^. This is likely explained by the lower oxidizing potency of the product, HOSCN. However, PXDN (0.5 μM), in the presence of 10 μM H_2_O_2_ and 500 μM SCN^-^, completely killed *P*. *aeruginosa* (S4 Fig). Neither truncated PXDN 29-250aa nor PXDN 251-609aa had the capacity to kill *P*. *aeruginosa* (Fig 3D). Similar capacity for *E*. *coli* killing was observed with FL-PXDN (Table 1). Interestingly, anti-lipid A antibody was able to significantly inhibit bacterial killing by PXDN (Fig 3E), consistent with the observation that PXDN binds to lipid A (Fig 2D). In addition, we evaluated bacterial killing of sera. Our data indicate that sera from PXDN-deficient mice is diminished capacity of bacterial killing, comparing with that from WT mice (Fig 3F), supporting a role of circulating PXDN in host defense.

**Table 1:** Bactericidal activities of PXDN, MPO and LPO. *E*. *coli* K12 was incubated in 50 mM phosphate buffer (pH 6.2) containing halide anion (Cl^-^, Br^-^ or I^-^), 10 μM H_2_O_2_, and indicated amount of hPx at 37°C for 1 h. Cell mixtures were plated on LB agar plates and incubated at 37°C overnight. The negative control experiment contained *E*.*coli* only. The CFUs were counted. The relative survival rate (%) was calculated as CFUs in the experimental group divided by those in the negative control. Data are representatives of at least three independent experiments.

	hPx (nM/heme)	Cl^-^ (100 mM)	Br^-^ (100 μM)	I^-^ (0.25 μM)	SCN^-^ (100 μM)
MPO	50	0.0%	0.0%	0.0%	56.7 ± 2.8%
	200	N/A	N/A	N/A	48.9 ± 0.9%
	1000	N/A	N/A	N/A	4.7 ± 3.2%
LPO	50	103.7 ± 8.6%	0.0%	98.1 ± 10.9%	104.7 ± 5.9%
	200	110.5 ± 15.4%	N/A	4.0 ± 0.7%	120.4 ± 6.9%
	1000	92.3 ± 8.6%	N/A	0.0%	82.6 ± 13.1%
PXDN	50	103.3 ± 7.6%	0.0%	62.5 ± 5.3%	71.8 ± 1.1%
	200	12.0 ± 1.8%	0.0%	0.0%	46.4 ± 2.2%
	1000	0.0%	0.0%	0.0%	32.7 ± 3.4%
0		118.7± 8.5%	89.6± 4.2%	97.7± 9.8%	102.0± 9.3%

We further examined whether PXDN is capable of killing GP bacteria, which lack LPS and do not directly bind PXDN (Fig 2E). We carried out the bacterial killing experiments in the presence of H_2_O_2_ and Cl^-^. Two different lots of recombinant FL-PXDN were utilized; both lots were unable to kill *S*. *aureus* and methicillin-resistant *Staphylococcus aureus* (MRSA), while their capacity to kill *E*. *coli* was maintained (Figs 3G and S5). Together, these data provide strong evidence that binding of PXDN to LPS of GN bacteria is essential for bacterial killing.

### PXDN secreted by lung epithelial cells kills bacteria

**Fig 4:**
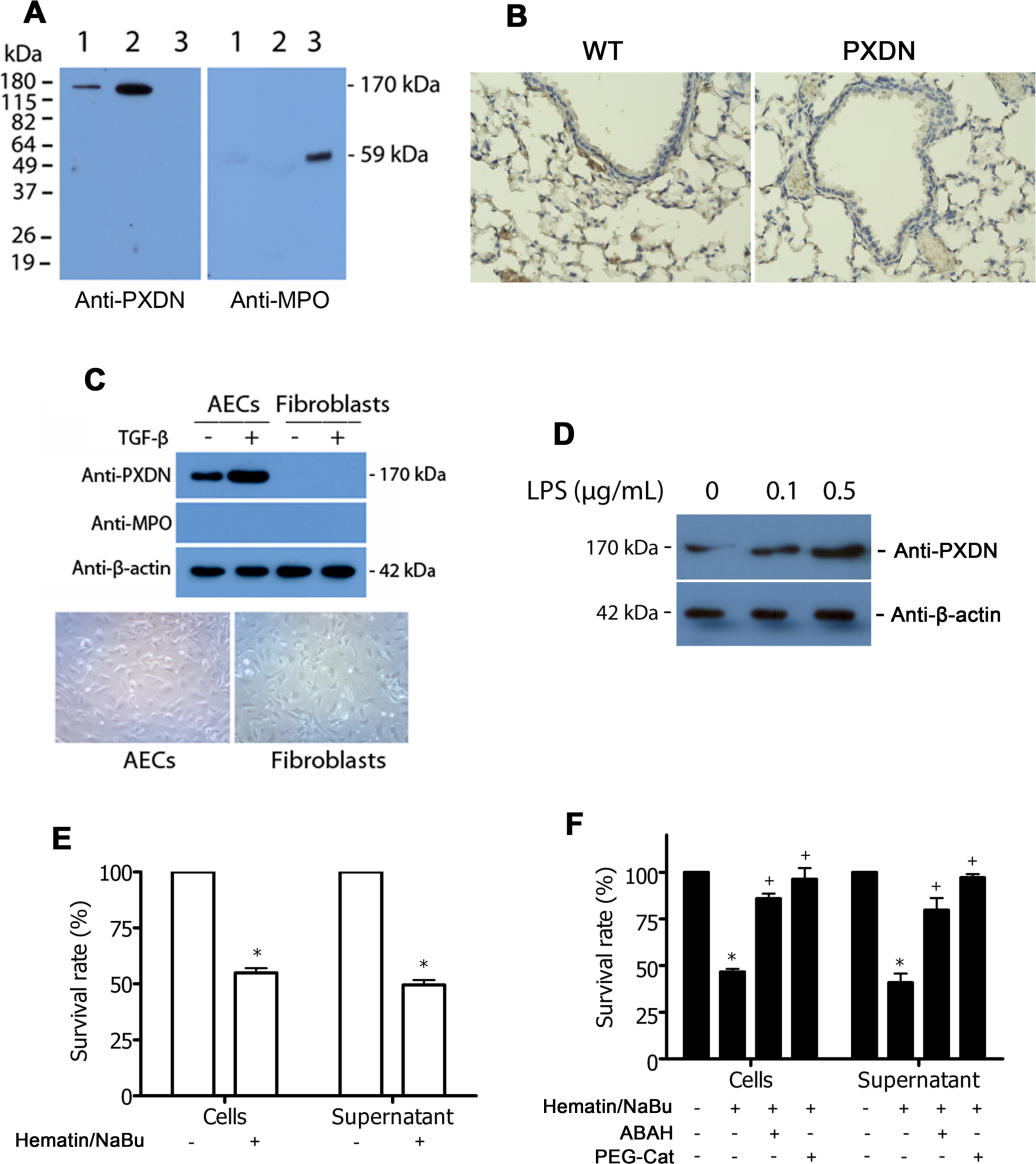
PXDN is expressed in the lung epithelium and contributes to bacterial killing. **(A)** Detection of PXDN in human bronchoalveolar lavage fluid (BALF). Right middle lobe BALF was collected and processed within 2 h. BALF samples were then centrifuged at 400 x *g* for 10 min at 4°C. BALF were concentrated by Centricon (~10x). Samples of BALF (1), positive control of human plasma containing ~100 ng of PXDN (2), and purified MPO (50 ng) (3) were subjected to immunoblotting using anti-PXDN or anti-MPO antibodies. **(B)** IHC of the lung. IHC of lung sections from WT and PXDN-deficient mice was performed using anti-PXDN antibody (1:600). Images were taken using BZ-X710 All-in-One Fluorescence Microscope. PXDN: dark brown. Magnification: 400x. **(C)** PXDN expresses in mouse primary lung type II alveolar epithelial cells (AECs). Upper panel showed immunoblot analysis of AECs and fibroblasts while lower panel showed phase contrast microscopy of AECs (left) and fibroblasts (right); magnification 100x. Cell lysates were subjected to conventional immunoblotting by using anti-PXDN antibody. β-actin was used as loading control. **(D)** LPS induces PXDN expression. AECs were induced by LPS as indicated. The cell lysates were subject to immunoblot analysis as in (**C**). **(E)** Mouse primary type II AECs mediate *P*. *aeruginosa* killing. AECs were grown in 12-well plate in DMEM with 10% FBS without antibiotics until 70% confluence. Cells were serum-starved for 16 h prior to bactericidal assays; cells were stimulated with/without TGF-β and hematin/NaBu. Cells and the overlying medium (supernatant) were separated and utilized for evaluation of PXDN-mediated bactericidal killing. 1 mL of fresh DMEM containing 10^4^ *P*. *aeruginosa* strain K and 10 μM H_2_O_2_ was added to AECs or supernatant. The mixture was incubated at 37°C for 1 h, and then plated on LB agar plates prior to overnight incubation at 37°C. The control group contained untreated AECs and *P*. *aeruginosa*. The relative survival rate was calculated as CFUs in the experimental group divided by those in the control group; *P< 0.001 *vs*. Control. **(F)** Inhibition of AEC-mediated *P*. *aeruginosa* killing by peroxidase inhibitors. The experiments were carried out as in “E” with addition of ABAH (broadly specific inhibitor of heme-containing peroxidases) or PEG-catalase (which reduces H_2_O_2_). *P< 0.001 *vs*. Control; ^+^*P* < 0.001 *vs*. hematin/NaBu. Data are representatives of three independent experiments.

The mammalian lung is endowed with multiple mechanisms of host defense that protect the mucosal epithelial barrier from pathogens, although a role for PXDN has not been established. We investigated the expression and distribution of PXDN in the mammalian lung and its potential role in lung host defense. First, high levels of PXDN were detected in bronchoalveolar lavage fluid (BALF) from normal human volunteers, whereas only trace amounts of MPO were detected (Fig 4A). Second, PXDN expressed in alveolar epithelial cells of WT mice, but not in PXDN-deficient mice (Fig 4B, dark brown). We further determined the expression of PXDN in type II alveolar epithelial cells (AECs). Primary type II AECs and fibroblasts were isolated from lungs of C57BL/6 mice using previously described methods [26]. PXDN was expressed in type II AECs, but not in fibroblasts (Fig 4C). PXDN expression in lung epithelial cells is also supported by the data of proteomic profiling and RNA-seq profiling (https://lungmap.net/). Interestingly, PXDN expression in AECs was induced by LPS in a dose-dependent manner (Fig 4D). We ascertained whether type II AECs are capable of PXDN-mediated *P*. *aeruginosa* killing. PXDN was expressed and activated by adding hematin (1 μg/mL) and sodium butyrate (NaBu, 5 mM), since the combination of hematin and NaBu induces PXDN expression and enhances PXDN activity [14]. After stimulation for 24 h, cells and medium were separated for evaluation of PXDN-mediated *P*. *aeruginosa* killing. AECs as well as the corresponding supernatants significantly induced *P*. *aeruginosa* killing (Fig 4E). 4-aminobenzoic acid hydrazide (ABAH), an inhibitor of hPx enzymes, and catalase, which reduces H_2_O_2_ to water, inhibited *P*. *aeruginosa* killing (Fig 4F). These studies demonstrate that lung epithelium is a source of PXDN which mediates H_2_O_2_-dependent bactericidal activity, a previously unrecognized host defense function of this enzyme.

### Deficiency of PXDN impairs bacterial killing and decreases survival in a murine model of GN bacterial pneumonia

**Fig 5:**
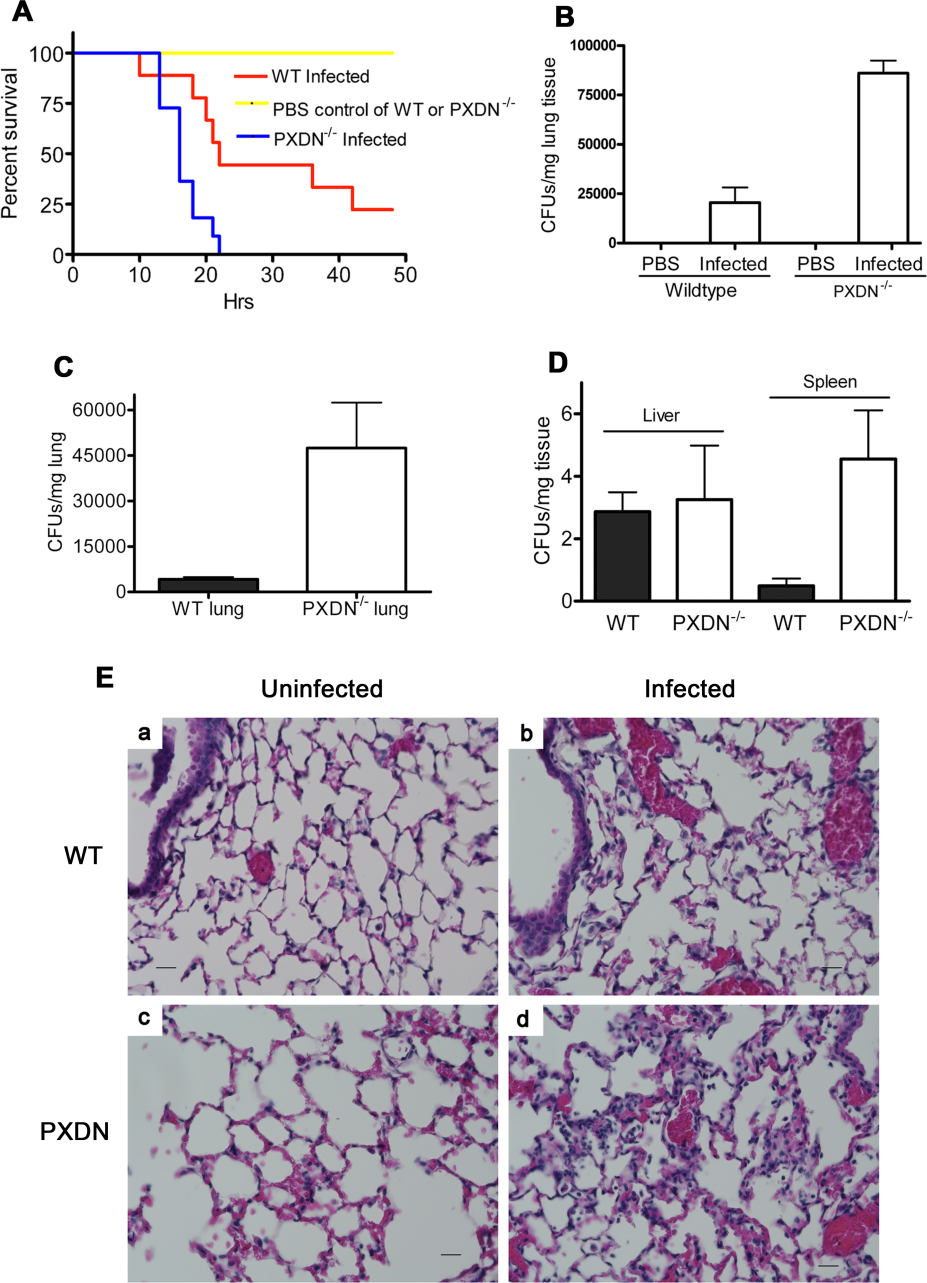
PXDN mutant mice reveal impairment in bacterial clearance during acute lung infection. **(A)** Decrease of relative survival rates. PXDN-deficient and C57BL/6 wild-type mice were intratracheally instilled with 7 x 10^6^ of *P*. *aeruginosa* strain K or PBS control. Relative survival rates were determined over a period of 48 hours; n = 9–11 per group, *P* = 0.0068. **(B)** Lung Bacterial burden. Mice were sacrificed at 20 hours; lungs were aseptically removed, weighed, and homogenized in PBS. Lung tissue suspension was serially diluted and plated on LB agar plates. After incubation at 37°C for 18 h, CFUs were counted and CFUs/mg tissue were calculated; n = 12 from 4 mice per group; one-way analysis of variance, *P* < 0.0001 for all comparisons. **(C and D)** Tissue burden on bacterial infection with sublethal dose was carried out by intratracheally infecting the mice with 3 x 10^6^ of *P*. *aeruginosa* strain K. After 20 h, lung, liver and spleen were taken as in (B) for detection of bacteria. n = 15–21 from 5–7 mice per group; P (lung) = 0.016; P (liver) = 0.843; P (spleen) = 0.014. **(E)** H&E staining of the lungs from uninfected and infected mice. Mice were infected as in (**C**) and the lungs were harvested at 20 h for preparation of staining. a. WT mouse, uninfected; b, WT mouse, infected; c. PXDN-deficient mouse, uninfected; d, PXDN-deficient mouse, infected. Magnification: 400x. Scale: 5μm.

To determine whether PXDN mediates critical host defense functions *in vivo*, we employed a murine model of GN bacterial pneumonia in wild-type and PXDN mutant mice (PXDN^mhdakta048^) [19]. *P*. *aeruginosa*, a leading cause of GN bacterial pneumonia in humans [27], were intra-tracheally injected into mice to induce acute lung infection. PXDN-deficient mice had markedly diminished survival, with 100% mortality at 24 h following infection while wild-type mice had 44% and 22% survival at 24 and 48 h, respectively (Fig 5A). Wild-type mice that survived acute infection at 48 h appeared to recover completely and remained healthy for several days. The high mortality in PXDN-deficient mice was associated with increased bacterial burden, as evidenced by CFUs of *P*. *aeruginosa* in lung tissues (Fig 5B). PBS treatment did not result in death in either strain of mice, and bacteria were absent in the lungs of these mice (Fig 5A and 5B). Intratracheal injection with sublethal dose of *P*. *aeruginosa* (3 x 10^6^ CFUs/mouse) showed increased bacterial burden in the lungs of PXDN-deficient mice (Fig 5C). In the liver and spleen, fewer bacterial CFUs were detected from both WT and PXDN-deficient mice; the bacterial number detected from the spleen from PXDN-deficient mice was significantly higher than that from WT mice (Fig 5D). The infected lungs of PXDN-deficient mice revealed more severe tissue injury and neutrophil infiltration, while uninfected lungs showed normal structure (Fig 5E). Together, these studies provide compelling data to support a critical host defense function of PXDN, specifically against GN bacterial pneumonia.

## Discussion

The lung is a uniquely vulnerable organ with a very thin, delicate epithelial lining, abundant blood flow, and a vast surface area. The lung resides at the interface of the body and environmental exposures to inhaled or aspirated pathogens. Thus, the lung is an important organ in host defense. Multiple layers of defense in the normal lung are involved in innate immune functions. Loss of one or more of these host defense mechanisms increases the susceptibility of the lung to infections. PXDN is a newly identified hPx, an enzyme family that plays an important role in host defense. Its physiological function is largely unknown, although recent studies implicate this gene in basement membrane synthesis [28, 29]. The present study, for the first time, identifies PXDN as a novel host defense enzyme in the lung with selectively for recognizing and directly killing GN bacteria. The N-terminus of PXDN, which contains five LRRs and four Ig domains, selectively binds to LPS while the C-terminus of PXDN containing the peroxidase domain kills GN bacteria *via* generation of hypohalous acids. An enzyme containing the molecular structure of both pattern-recognition domain and scavenger domain suggests evolutionary conservation of a dual-function protein capable of both pathogen recognition and killing, expanding our current view of innate immunity.

The original theory of innate immune pattern recognition is based on the interaction between host PRRs and specific PAMPs, and the activation of downstream signaling events and host defense mechanisms [4, 5]. On the other hand, many effectors including complement system, antimicrobial peptides, and lysozymes may bind to important bacterial molecules and directly kill pathogens. For example, neutrophils possess bactericidal permeability-increasing protein (BPI) in their azurophilic granules. BPI is structurally related to LPS binding protein, and avidly binds LPS in GN organisms to directly kill them by compromising membrane integrity [30]. Peptidoglycan recognition proteins (PGRPs) are innate immune molecules present in insects, mollusks, echinoderms, and vertebrates. Mammals have four PGRPs. One mammalian PGRP, PGLYRP-2, is an *N*-acetylmuramoyl-L-alanine amidase that hydrolyzes bacterial peptidoglycan and reduces its pro-inflammatory activity. The three remaining PGRPs kill bacteria by interacting with cell wall peptidoglycan [31]. Our data support the uniqueness of PXDN, among the known mammalian hPx’es, based on its structural characteristics that allows for its dual-function in pathogen recognition and killing.

PXDN is found in circulating plasma at a concentration of 1.1 ± 0.6 μM in humans and 2.6 ± 0.6 μM in mouse, approximately ~1000-fold higher than MPO [20]. Although its peroxidase activity is ~5–10% of MPO activity [14], the net activity of PXDN may be 50–100 folds higher than MPO in plasma. The high expression of PXDN in alveolar epithelium and in bronchoalveolar lavage fluid suggests that this enzyme may mediate critical host defense functions and mucosal immunity. The ability of PXDN to directly bind GN bacterial pathogens allows for more targeted activation and killing without collateral damage to surrounding tissues.

The concept of targeted killing is further supported by the finding that LPS induces PXDN activation, a phenomenon has not been reported for other members of the hPx family. Our data reveals that LPS is able to activate PXDN by ~4-fold. Thus, the bactericidal activity of PXDN to GN bacteria is great increased in a selective and targeted manner. The mechanism of activation of PXDN by LPS may be due to LPS-mediated conformation changes of PXDN since the appropriate conformation is critical for the catalytic activity of hPx enzymes [8]. The activation of PXDN by LPS has important implications for enhanced bactericidal activity, while limiting damage to host tissues.

*P*. *aeruginosa* is a pathogen responsible for a variety of severe infections, including acute lower respiratory tract infections in both immunocompetent and immunocompromised hosts, as well as chronic respiratory infections in select patient populations such as those with cystic fibrosis [32]. High incidence, infection severity and increasing resistance characterize *P*. *aeruginosa* infections, highlighting the need for new therapeutic options. Our findings of PXDN killing of GN bacteria, including *P*. *aeruginosa*, support therapeutic interventions involving PXDN to augment host defense against such infections.

In summary, PXDN is a novel host defense enzyme in the lung with dual function in pathogen recognition and killing. Further studies are required to determine its role in systemic immunity. The unique structural and functional characteristic of PXDN expands our current understanding of mucosal innate immunity, and has important implications for novel therapeutic strategies.

## Materials and methods

### Animals

C57BL/6 and PXDN^mhdakta048^ mutant mice (male and females, 8–12 week-old) were used in the study. PXDN^mhdakta048^ mice were in C57BL/6 background [19]. Unless otherwise stated, mice were fed with normal chow diet.

### Ethics statement

The protocol of animal study was approved by the Institutional Animal Care and Use Committee of the University of Alabama at Birmingham with approval number 20223. The animal care and use are adhered to the regulations and guidelines of International Association of Assessment and Accreditation of Laboratory Animal Care, Office of Laboratory Animal Welfare and the United State Department of Agriculture.

### Bacteria and PXDN binding assay

Recombinant FL-PXDN, PXDN 29-250aa or PXDN 251-609aa was added into 100 μL PBS containing 4 x 10^8^ live *P*. *aeruginosa* strain K or *E*. *coli* K12. The mixture was incubated at RT for 1 h. Bacteria were spun down at 3099 x g for 5 min. The cells were washed twice by 5 x initiating volume of PBS. Bacteria were lysed in 2 x SDS-PAGE loading buffer and the lysates were subject to immunoblot analysis using anti-His antibody. Protein bands were visualized by chemiluminescence. Negative controls contained only PXDN or bacteria. Quantitative analysis was carried out by using ImageJ software (The National Institute of Health). In some experiments, LPS-deficient *E*. *coli* stains were utilized. LPS-deficient *E*. *coli* strains and their parent strain K12 BW25113 were from The Coli Genetic Stock Center at Yale University. In bacteria and plasma PXDN binding assay, 4 x 10^8^ *E*. *coli* or *P*. *aeruginosa* in 50 μL PBS were mixed with 50 μl of human plasma. Control groups were cells or plasma alone. In some experiments, lipid A (Sigma-Aldrich Cat. #L5399) was used. In brief, indicated amount of lipid A was mixed with 150 nM of recombinant PXDN in 600 μL PBS. The mixture was shacked at RT for 15 min. Then 2 x 10^8^ live *E*.*coli* K12 cells were added into the mixture and incubated at RT for additional 30 min. Bacterial pellets were obtained and subject to immunoblot analysis.

### Surface plasmon resonance (SPR)

Analyses were carried out using a Biacore-T200 instrument (GE-Healthcare) at 22°C in PBS. Recombinant PXDN 29-250aa or PXDN 251-609aa (50 μg/mL) were captured onto an NTA Sensor Chip (GE-Healthcare), respectively. LPS (2 μM) was injected over each surface, as well as over a blank surface. Full kinetics was carried out by flowing a serial concentration (range from 0.25 to 10.0 μM) of LPS over the chip. Binding data (Ka, Kd and KD) were collected and analyzed by using the BIAevaluation software (Biacore). All measurements were conducted in triplicate. Rate constants of SPR were calculated as following.

$$\begin{array}{l} \;ka\\ A+B↔AB\\ \;kd \end{array}$$

Association rate: d[AB]/dt = ka·[A]·[B]

Dissociation rate: -d[AB]/dt = kd·[AB]

Equilibrium dissociation constant: KD = kd/ka = [A]·[B]/[AB]

### 3,3’,5,5’-Tetramethylbenzidine (TMB) oxidation assay

hPx as indicated was added into 100 μL of TMB solution (TMB Liquid Substrate System, Sigma-Aldrich), which contains H_2_O_2_. Reaction mixture was incubated at RT for 30 min. TMB oxidation was recorded at absorbance 650 nm. In some experiments, LPS (Sigma-Aldrich Cat #L2880, from *E*. *coli* 055:B5) or GN bacteria were mixed with 400 nM/heme of recombinant PXDN at RT for 30 min. 20 μL of mixture was added into 100 μL TMB solution. After 30 min, absorbance at 650 nm was recorded.

### *In vitro* killing of bacteria by PXDN

*P*. *aeruginosa* strain K and *E*. *coli* K12 cells were incubated in 50 mM phosphate buffer (pH 6.2) containing 140 mM NaCl, 10 μM H_2_O_2_, and indicated amounts of PXDN at 37°C for 1 h. Cell mixtures were plated on LB agar plates, followed by incubation at 37°C overnight. In control experiments, only H_2_O_2_ (10 μM) or Cl^-^ (140 mM) was present. The CFUs were counted and relative survival rates were calculated as CFUs in the experimental group divided by CFUs in the control group. Other halide anions (bromide, iodide and thiocyanate) in addition to Cl^-^ were used as indicated. In some experiments, anti-lipid A antibody was used for inhibition of bacterial killing by PXDN. GP bacteria were also utilized in some bacterial killing experiments.

### Bactericidal activity of serum

100 μL reaction mixtures contained 50 μL serum from C57BL/6 or PXDN-deficient mouse and 50 μL PBS containing *P*. *aeruginosa* and 50 μM H_2_O_2_. After incubation at 37° for 1h, the mixture was plated on LB agar plates and incubated at 37° for overnight. Colonies were counted.

### L-012 oxidation assay

Chemiluminescent dye L-012 is a sensitive substrate for measuring heme-containing peroxidase activity. It generates chemiluminescence once oxidation. In present study, 20 μM (final concentration) was added into 100 μL of bacterial suspension containing bound hPx and 20 μM of H_2_O_2_. Chemiluminescent light at 450 nm was immediately recorded by a luminometer (Molecular Devices, Sunnyvale, CA).

### Lung tissue staining

Mouse lung tissue was harvested and fixed in 10% formalin. The tissue sections (5 μm) were prepared, and Haemotoxylin and Eosin (H&E) staining was performed at the Comparative Pathological Laboratory at the University of Alabama at Birmingham. The Conventional immunohistochemistry (IHC) was carried out by using anti-PXDN antibody (1:600). Images were taken using BZ-X710 All-in-One Fluorescence Microscope (Keyence Corporation of America, Itasca, IL, USA).

### Separation of mouse primary lung type II alveolar epithelial cells (AECs)

Mouse primary lung type II AECs were isolated as described in [26] with slight modification. In brief, 4–5 mice were euthanized with CO_2_. Blood was exsanguinated by clipping abdominal aorta. Trachea and lungs were carefully exposed and lungs were perfused through puncture of right ventricle with 10 mL of sterile PBS until lungs clear of blood. 18G catheter was inserted into trachea. 1 mL of dispase II (5 U/mL) per mouse was instilled into the lung; then 1% warm low melting agarose was instilled. Lungs were carefully removed and incubated in dispase II solution for 45 min. Lung tissue was minced with scissors until the consistency of jelly. Minced lungs were incubated with 5 mL per mouse of DNase I (42 U/mL)/DMEM solution for 10 min; then the suspension was filtered through successive filters (100 μm, 35 μm and 15 μm). Biotin-conjugated anti-CD32/16 (BD Biosciences, Cat. #BD553143, 15.6 μl per mouse) and Biotin-conjugated anti-CD45 (BD Biosciences, Cat. #BD553078, 36 μl per mouse) were added into the filtered suspension and incubated at 37°C for 30 min with gentle shake. The filtered suspension was centrifuged and cells were re-suspended in complete media (5 mL/mouse). 1 mL of streptavidin-magnetic beads (Promega, Cat. #8452) was added into the cells and the mixture was incubated for 30 min. The media containing unbound cells were carefully removed and placed into culture dish. The cells were then incubated at 37°C overnight. Next day, the media containing non-adherent cells were transferred into 50 mL-conical tube. The remaining adherent cells were fibroblast. The cell suspension in 50-mL tube was centrifuged at 344 x g for 10 min at 4°C. The cell pellet was re-suspended in complete media. Cells were placed into fibrinogen coated plates. These cells were type II alveolar cells, generally with ~95% purity, evaluated using anti-surfactant Protein C antibody. All steps were carried out aseptically.

### Immunoblot analysis

The conventional immunoblotting assay was carried out using anti-PXDN affinity-purified polyclonal antibody (against residues 49–63 of human PXDN) [20], anti-MPO polyclonal antibody (CALBIOCHEM, Cat #475915) or anti-His antibody (Qiagen). BALF samples were centrifuged at 400 x *g* for 10 min at 4°C. Resultant supernatants were portioned and stored at -80°C for analysis. BALF was concentrated by Centricon (~10x). Human plasma and purified MPO (Cat. #MY167, Elastin Products Company, Owensville, MO) were as positive controls for PXDN and MPO, respectively. In some experiments, the primary lung type II AECs were stimulated with 2 ng/mL of TGF-β or LPS (0.1 or 0.5 μg/mL) for 24 hrs. Cell lysates were subject to conventional immunoblotting by using anti-PXDN or anti-MPO antibody. β-actin was used as loading control.

### PXDN-mediated bacterial killing of primary lung type II AECs

AECs were cultured in 12-well plate in DMEM (Life Technologies, Inc.) supplemented with 10% FBS (Life Technologies, Inc.) without antibiotics. Cells were incubated at 37°C in 5% CO_2_ until 70% confluence. Cells were serum-starved for 16 hrs. Some cells were induced by addition of TGF-β1 (2 ng/mL) or NaBu (5 mM)/hematin (1 μg/mL) for 24 h to increase the PXDN expression. In some experiments, ABAH (100 μM) or catalase-polyethylene glycol (PEG-cat, 200 U/mL) was added. Cells and medium were separated for evaluation of the bacterial killing, respectively. 100 μL supernatant of medium were incubated with 4 x 10^4^ *P*. *aeruginosa* cells containing 10 μM H_2_O_2_ at 37°C for 1 h. AECs (~5 x 10^5^) were added 1 mL fresh DMEM plus 4 x 10^4^ *P*. *aeruginosa* cells and 10 μM H_2_O_2_ and incubated at 37°C for 1 h. The mixture was plated on LB agar plates followed by incubation at 37°C overnight. The CFUs were counted and relative survival rate was calculated as CFUs in the experimental group divided by CFUs in the control group.

### Acute lung infection model

*P*. *aeruginosa* strain K (a gift *of Dr*. Jean-Francois Pittet at the Department of Anesthesiology, UAB) was used for acute infections. Inoculums for mouse infections will be prepared as previously described with modification [33]. In Brief, bacteria from LB agar plate were inoculated in 5 mL of LB broth at 37°C with shaking (175 rpm). After 16–18 h of incubation at 37°C, the stationary-phase bacteria were pelleted, washed 3 times with 15 mL of sterile in PBS, and re-suspended in 3 mL sterile PBS. This stock will be diluted in sterile PBS to give an appropriate titer. Female and male C57BL/6 mice or PXDN mutant mice at 8–12 week-old (9-11/group) were used. The mice were briefly anaesthetized with ketamine-xylazine (100 mg/kg ketamine and 6 mg/kg xylazine) *via* intraperitoneal. The mouse was laid on a board with head elevated at 45°. 30 μL of PBS containing 3.0 x 10^6^ or 7.0 x 10^6^ CFUs of *P*. *aeruginosa* strain K was intratracheally instilled with a 29 G gauge needle. Mice were allowed to recover for 30–60 min prior to being returned to the cage.

### Bacterial burden

50–100 mg of lung, liver or spleen was aseptically removed from mice with cervical dislocation at 20 h after instillation. The tissue was weighed and homogenized for bacteria clearance analysis. Homogenized tissue was washed with 4 mL sterile PBS. The tissue suspension was filtered through 100 μm Nylon mesh (Fisher Scientific, Cat #22363549) to remove tissue debris. The filtered suspension was centrifuged at 3099 x g at 4°C for 10 min to pellet the bacteria. The pellet was re-suspended in sterile PBS (10 μL/mg tissue). The bacterial resuspension was serial dilution. 50 μL of sample was plated on LB agar plates (triplicate) and the plates were incubated at 37°C for 18 h. Bacterial colonies were counted for analysis.

### Statistical analysis

Data were shown as means ± SD, unless otherwise indicated. Quantitative variables were compared by means of Student's *t*-test for two groups or ANOVA for multiple groups. A value of P < 0.05 was considered significant.

## Supporting information

S1 Fig Control protein, ceruloplasmin, does not bind to *P*. *aeruginosa*. Experiment was carried out as in Fig 1D. 4 x 10^8^ of *E*. *coli* K12 or *P*. *aeruginosa* strain K in 50 μL PBS was added to 50 μL human plasma. The mixtures were incubated at 37°C for 1 h. Bacteria suspensions were spun down at 3099 x g for 5 min, and then washed twice with 500 μL PBS. Samples were subjected to immunoblotting using anti- PXDN or anti-ceruloplasmin antibodies. “C” represents positive control of 1 μL plasma (containing 200 ng of PXDN and 200–600 ng of ceruloplasmin) directly loaded onto the gel.
(TIF)

S2 Fig Living *E*. *coli* K12 or *P*. *aeruginosa* strain K does not significantly oxidize TMB. Experiments were similar to Fig 3B and 3C. Indicated number of bacteria was added into TMB solution. Absorbance at 650 nm was measured. “V” is recombinant FL-PXDN as positive control. *P < 0.0001 *vs*. experimental group with 10^7^ bacteria.
(TIF)

S3 Fig GN bacteria do not activate MPO and LPO. TMB oxidation assay was carried out similar to Fig 3B and 3C. Indicated number of *E*. *coli* was added into TMB solution containing 50 nM MPO (A) or 50 nM LPO (B). Absorbance at 650 nm was measured. *P > 0.05 *vs*. control; #P < 0.05 *vs*. control.
(TIF)

S4 Fig PXDN completely kills *P*. *aeruginosa* in 500 μM SCN^-^. The experiment was similar to that in Fig 3D. *P*. *aeruginosa* strain K suspensions were incubated in 50 mM phosphate buffer (pH 6.2) containing 500 nM FL-PXDN, 10 μM H_2_O_2_, and 500 μM KSCN) at 37°C for 1 h. Cell mixtures were plated on LB agar plates and incubated at 37°C overnight. The CFUs were counted, and relative survival rates were calculated. n = 3, *P* = 0.0004.
(TIF)

S5 Fig PXDN does not kill GP bacteria. The bactericidal experiments were carried out as in Fig 3G in the presence of H_2_O_2_ and NaCl. Photographs of petri dishes were taken. Data are representatives of three plates. Two lots of recombinant PXDN were used. *E*. *coli* was used as positive control.
(TIF)
