# Supplementary figures and images for: Peroxidasin contributes to lung host defense by direct binding and killing of gram-negative bacteria
Source: PLoS Pathog. 2018 May 18;14(5):e1007026. doi: 10.1371/journal.ppat.1007026 (PMC5979044; doi:10.1371/journal.ppat.1007026)

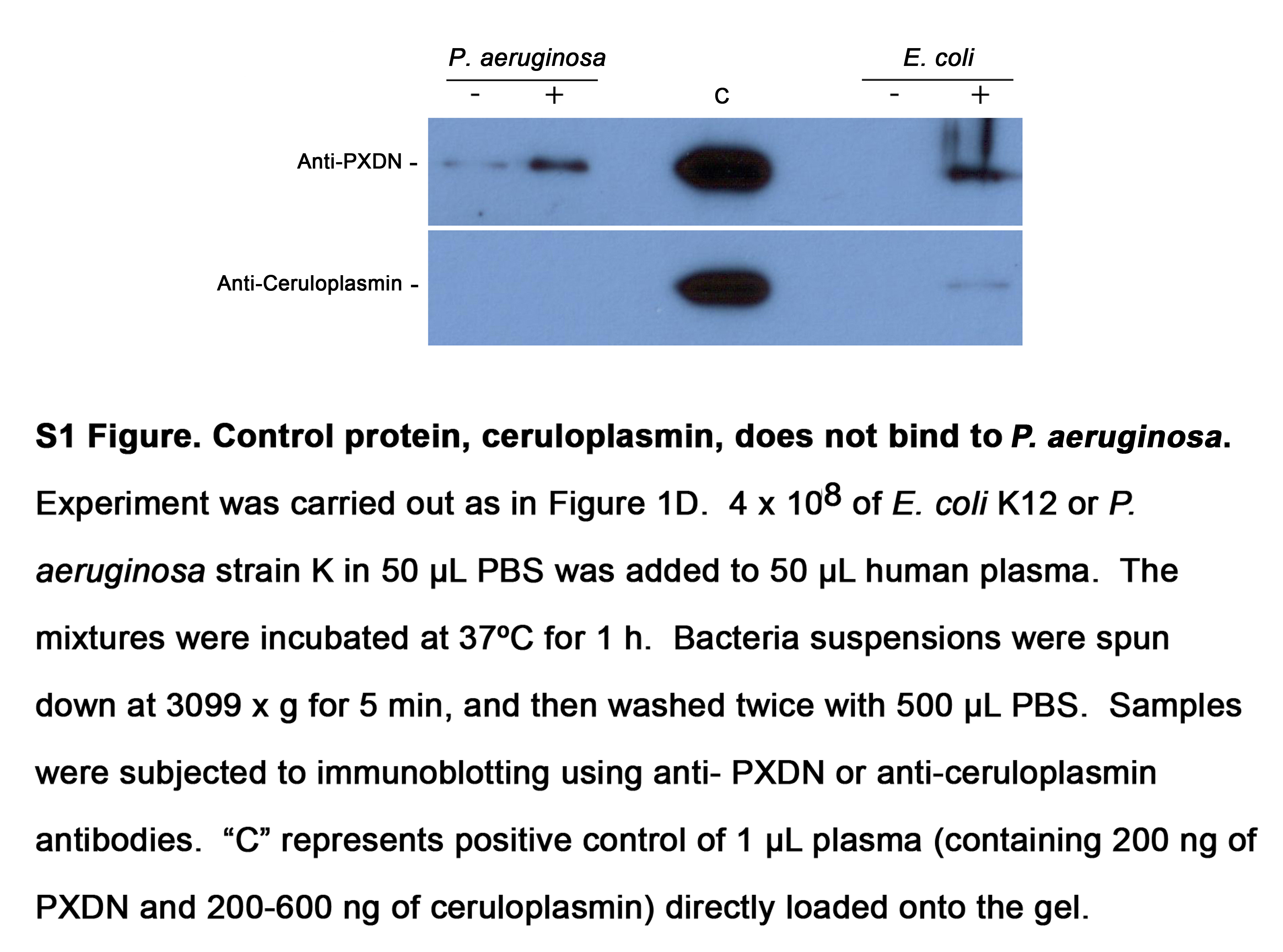

Supplement: S1 Fig — Experiment was carried out as in Fig 1D. 4 x 10^8^ of E. coli K12 or P. aeruginosa strain K in 50 μL PBS was added to 50 μL human plasma. The mixtures were incubated at 37°C for 1 h. Bacteria suspensions were spun down at 3099 x g for 5 min, and then washed twice with 500 μL PBS. Samples were subjected to immunoblotting using anti- PXDN or anti-ceruloplasmin antibodies. “C” represents positive control of 1 μL plasma (containing 200 ng of PXDN and 200–600 ng of ceruloplasmin) directly loaded onto the gel. (TIF) [file ppat.1007026.s001.tif]

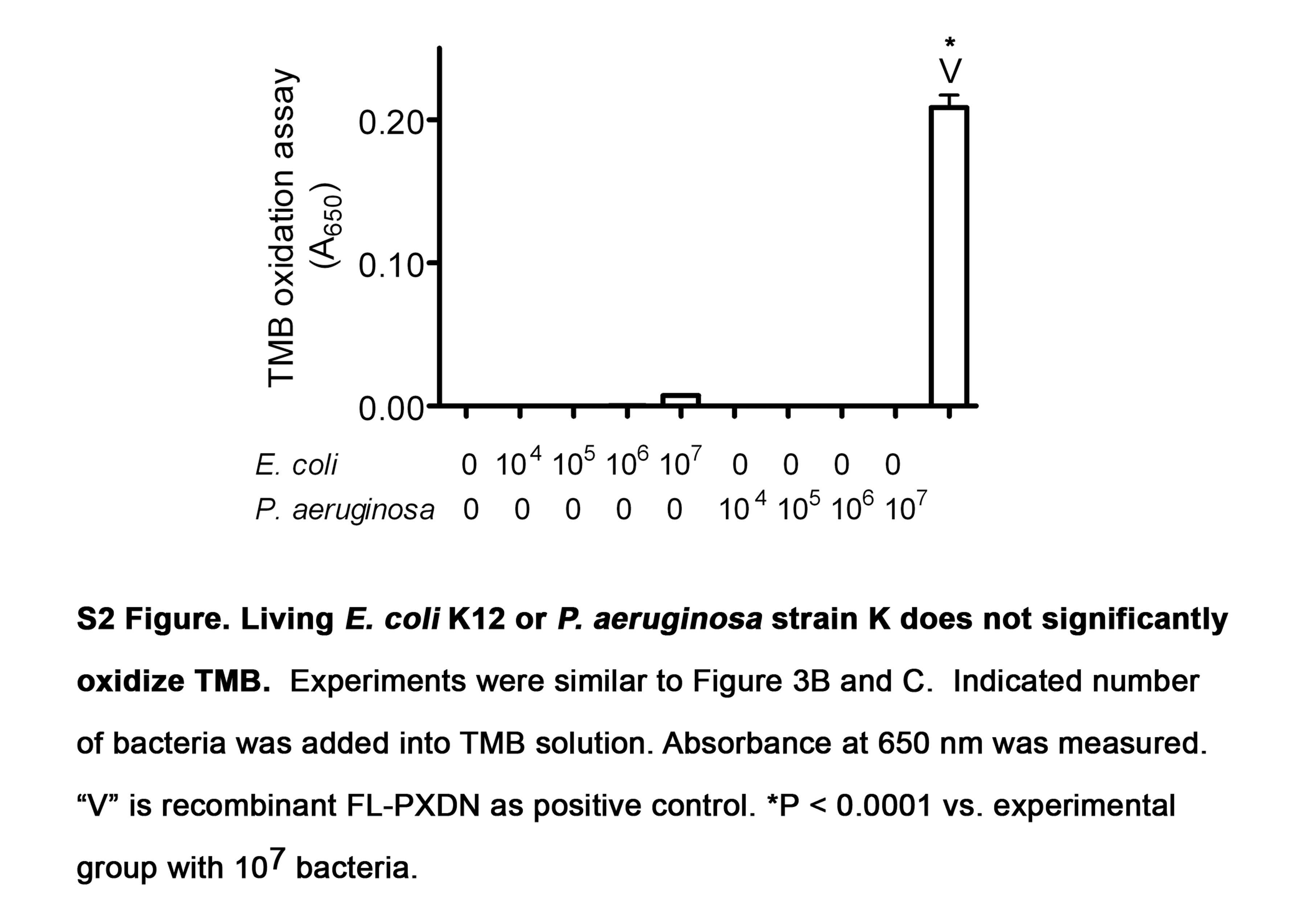

Supplement: S2 Fig — Experiments were similar to Fig 3B and 3C. Indicated number of bacteria was added into TMB solution. Absorbance at 650 nm was measured. “V” is recombinant FL-PXDN as positive control. *P < 0.0001 vs. experimental group with 107 bacteria. (TIF) [file ppat.1007026.s002.tif]

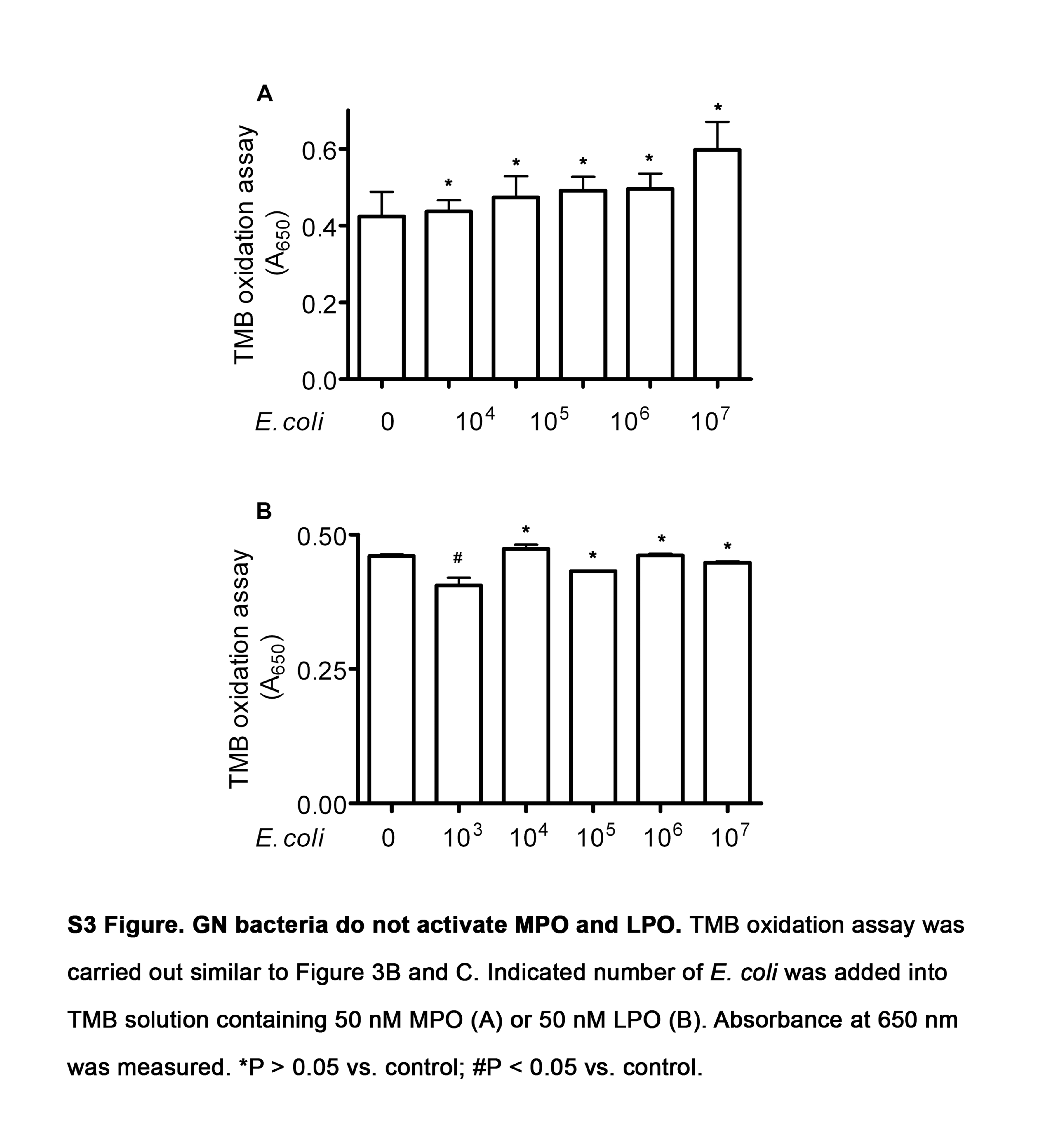

Supplement: S3 Fig — TMB oxidation assay was carried out similar to Fig 3B and 3C. Indicated number of E. coli was added into TMB solution containing 50 nM MPO (A) or 50 nM LPO (B). Absorbance at 650 nm was measured. *P > 0.05 vs. control; #P < 0.05 vs. control. (TIF) [file ppat.1007026.s003.tif]

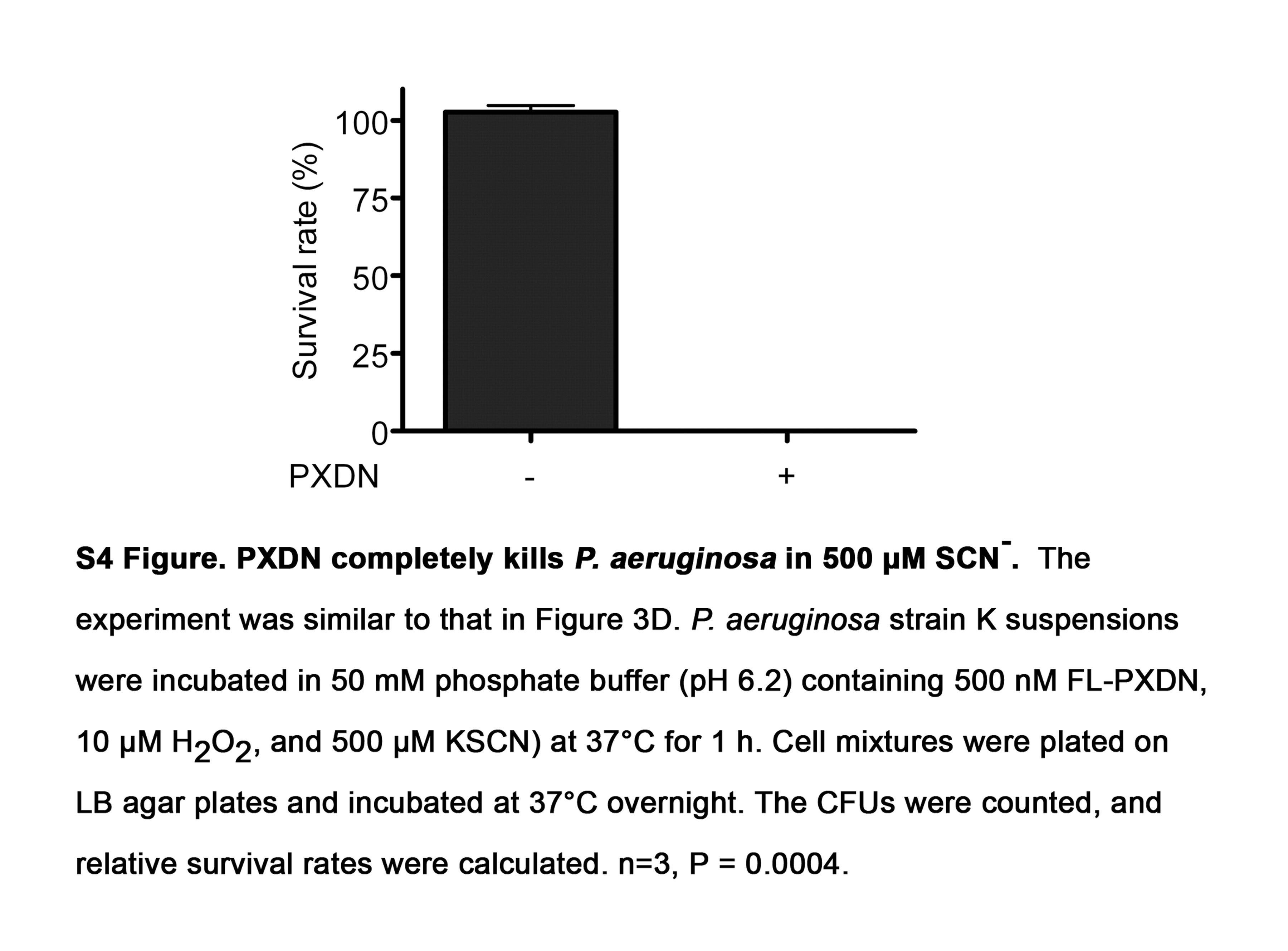

Supplement: S4 Fig — The experiment was similar to that in Fig 3D. P. aeruginosa strain K suspensions were incubated in 50 mM phosphate buffer (pH 6.2) containing 500 nM FL-PXDN, 10 μM H2O2, and 500 μM KSCN) at 37°C for 1 h. Cell mixtures were plated on LB agar plates and incubated at 37°C overnight. The CFUs were counted, and relative survival rates were calculated. n = 3, P = 0.0004. (TIF) [file ppat.1007026.s004.tif]

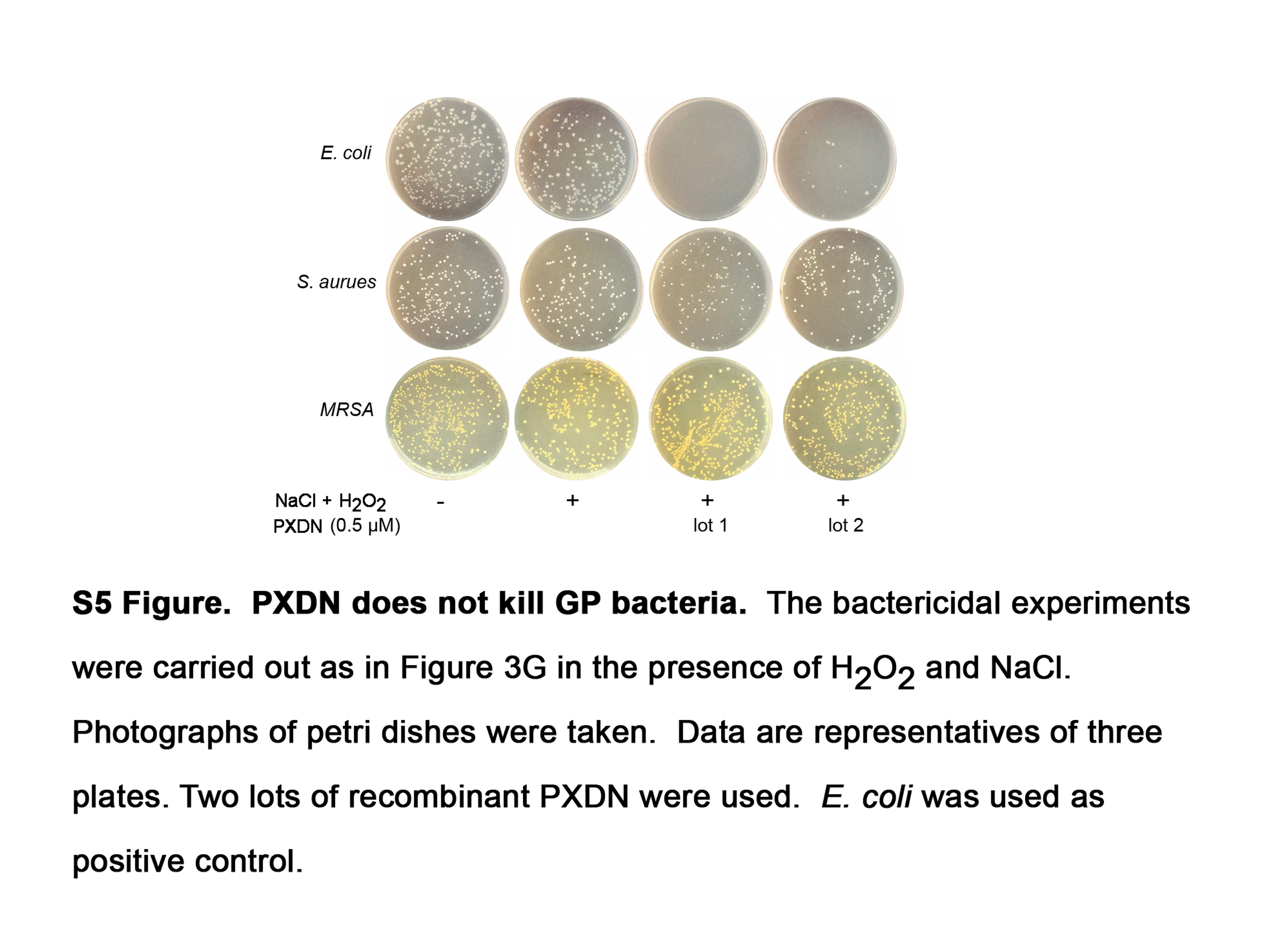

Supplement: S5 Fig — The bactericidal experiments were carried out as in Fig 3G in the presence of H2O2 and NaCl. Photographs of petri dishes were taken. Data are representatives of three plates. Two lots of recombinant PXDN were used. E. coli was used as positive control. (TIF) [file ppat.1007026.s005.tif]
